# Improving the Generalizability and Performance of an Ultrasound Deep Learning Model Using Limited Multicenter Data for Lung Sliding Artifact Identification

**DOI:** 10.3390/diagnostics14111081

**Published:** 2024-05-22

**Authors:** Derek Wu, Delaney Smith, Blake VanBerlo, Amir Roshankar, Hoseok Lee, Brian Li, Faraz Ali, Marwan Rahman, John Basmaji, Jared Tschirhart, Alex Ford, Bennett VanBerlo, Ashritha Durvasula, Claire Vannelli, Chintan Dave, Jason Deglint, Jordan Ho, Rushil Chaudhary, Hans Clausdorff, Ross Prager, Scott Millington, Samveg Shah, Brian Buchanan, Robert Arntfield

**Affiliations:** 1 Department of Medicine, Western University, London, ON N6A 5C1, Canada; rchaud@uwo.ca; 2 Faculty of Mathematics, University of Waterloo, Waterloo, ON N2L 3G1, Canada; delaneygracesmith22@gmail.com (D.S.); h349lee@uwaterloo.ca (H.L.); 3 Faculty of Engineering, University of Waterloo, Waterloo, ON N2L 3G1, Canada; amir.roshankar@uwaterloo.ca (A.R.); b384li@uwaterloo.ca (B.L.); 77faraz77@gmail.com (F.A.); m2arahman@uwaterloo.ca (M.R.);; 4 Division of Critical Care Medicine, Western University, London, ON N6A 5C1, Canada; john.basmaji@lhsc.on.ca (J.B.); cdave@qmed.ca (C.D.); ross.prager@lhsc.on.ca (R.P.); robert.arntfield@gmail.com (R.A.); 5 Schulich School of Medicine and Dentistry, Western University, London, ON N6A 5C1, Canada; jtschirhart2024@meds.uwo.ca (J.T.); adurvasula2025@meds.uwo.ca (A.D.); cvannelli2025@meds.uwo.ca (C.V.); 6 Independent Researcher, London, ON N6A 1L8, Canada; aford3532@gmail.com; 7 Faculty of Engineering, Western University, London, ON N6A 5C1, Canada; bennettjlvb@gmail.com; 8 Department of Family Medicine, Western University, London, ON N6A 5C1, Canada; jho2021@meds.uwo.ca; 9 Departamento de Medicina de Urgencia, Pontificia Universidad Católica de Chile, Santiago 8331150, Chile; hjclausd@uc.cl; 10 Department of Critical Care Medicine, University of Ottawa, Ottawa, ON K1N 6N5, Canada; scottjmillington@gmail.com; 11 Department of Medicine, University of Alberta, Edmonton, AB T6G 2R3, Canada; samveg.shah@medportal.ca; 12 Department of Critical Care, University of Alberta, Edmonton, AB T6G 2R3, Canada; brian.buchanan24@gmail.com

**Keywords:** artificial intelligence, deep learning, explainability, generalizability, lung ultrasound, lung sliding, multicenter, pneumothorax, POCUS, ultrasound

## Abstract

Deep learning (DL) models for medical image classification frequently struggle to generalize to data from outside institutions. Additional clinical data are also rarely collected to comprehensively assess and understand model performance amongst subgroups. Following the development of a single-center model to identify the lung sliding artifact on lung ultrasound (LUS), we pursued a validation strategy using external LUS data. As annotated LUS data are relatively scarce—compared to other medical imaging data—we adopted a novel technique to optimize the use of limited external data to improve model generalizability. Externally acquired LUS data from three tertiary care centers, totaling 641 clips from 238 patients, were used to assess the baseline generalizability of our lung sliding model. We then employed our novel Threshold-Aware Accumulative Fine-Tuning (TAAFT) method to fine-tune the baseline model and determine the minimum amount of data required to achieve predefined performance goals. A subgroup analysis was also performed and Grad-CAM++ explanations were examined. The final model was fine-tuned on one-third of the external dataset to achieve 0.917 sensitivity, 0.817 specificity, and 0.920 area under the receiver operator characteristic curve (AUC) on the external validation dataset, exceeding our predefined performance goals. Subgroup analyses identified LUS characteristics that most greatly challenged the model’s performance. Grad-CAM++ saliency maps highlighted clinically relevant regions on M-mode images. We report a multicenter study that exploits limited available external data to improve the generalizability and performance of our lung sliding model while identifying poorly performing subgroups to inform future iterative improvements. This approach may contribute to efficiencies for DL researchers working with smaller quantities of external validation data.

## 1. Introduction

Deep learning (DL) has proven superior to standard computer vision techniques for various medical imaging tasks including disease classification, segmentation, and image enhancement across several imaging modalities such as CT, MRI, ultrasound, and histological images [1]. The power of DL in medical imaging stems from automated feature extraction of complex images by leveraging large datasets [2]. As the pace of DL research for medical imaging accelerates, an abundance of single-center trained models are being developed [3,4,5]. The challenges of obtaining and working with high-quality, external imaging data often stunt models from gaining the necessary validation required for eventual clinical deployment. Furthermore, even when external data are available, performance degradation is routinely observed when models are tested against external datasets [6,7,8,9]. Point-of-care ultrasound data presents additional unique challenges including various manufacturers, scanning presets, and probe types used based on individual institutional practices. Thus, there is an urgency to optimize the use of external datasets that serve both as validation data, as well as a substrate for fine-tuning to maximize performance on holdout data.

Presently, the issue of poor generalizability in the face of scarcely available labelled medical data is addressed by utilizing transfer learning techniques and data augmentation [10]. Initializing model weights using datasets such as ImageNet [11] leverages learned features to reduce inference times and improve generalizability. However, features learned from natural images may not necessarily reflect medical images. In fact, images from ImageNet demonstrate the most dissimilarity to point-of-care ultrasound images compared to other medical imaging modalities [12]. Alzubaidi et al. [13] address this problem by investigating in-domain transfer learning in which model weights are initialized using related medical images (skin cancer) before fine-tuning on a target domain (diabetic foot ulcers). They were able to demonstrate improved model performance.

Significant train and test set performance differences are observed with single random splitting of datasets, which is exacerbated in smaller datasets [14]. K-fold cross validation is a popular resampling method that maximizes the use of a dataset splitting by fitting and averaging the performance of k models [15]. Despite its purported benefits of reducing overfitting, k-fold cross validation has been demonstrated to introduce biases with small sample sizes [16]. To address this, methods such as nested cross validation have been investigated and exhibit more robust performance by uncoupling the process of hyperparameter optimization and model selection [16].

Our group previously developed DL classifiers to evaluate lung ultrasound (LUS) clips for several respiratory pathologies, including the detection of lung sliding [17,18]. The lung sliding artifact is used to assess for the potentially life-threatening condition pneumothorax (PTX) and presents an opportunity for decisive clinical utility if its detection can be automated and validated [19,20,21,22]. Our previously developed lung sliding classifier achieved desirable performance on a 540-clip holdout set (0.935 sensitivity, 0.873 specificity, and 0.973 AUC), improving upon existing work that focused on animal models [23,24] and small, homogeneous human datasets [25]. Although our model was trained using a comparably large dataset for LUS research, the data used for training was sourced from a single institution. At this time, the performance of our model on LUS clips acquired at other institutions is unknown.

We present a multicenter study that aims to: (1) investigate a new approach for dataset splitting to optimally leverage scarcely available external data to improve the generalizability of our lung sliding classifier and (2) utilize important metadata to identify poorly performing subgroups that may inform future iterative improvements. Our contributions to the field offer an alternative strategy, in addition to the traditional methods of data augmentation, transfer learning, and k-fold cross validation, with variable-sized dataset splitting to improve the generalizability of deep learning models that have limited availability of external datasets.

## 2. Materials and Methods

Our project received research ethics board approval from Western University (REB 116838) on 28 January 2021.

### 2.1. Dataset Description and Preparation

The external dataset described in this work was collected from three partner institutions located in Edmonton, Canada (D_462_); Santiago, Chile (D_117_); and Ottawa, Canada (D_62_). The nomenclature (D_x_) is based on the number of clips x that each institution contributed to the combined dataset. The creation of this database and our LUS labelling workflow have been detailed previously [17]. Given the paucity of absent lung sliding clips at some institutions, all datasets were combined to form a composite external dataset (D_all_) to be used to fine-tune the model originally trained on data collected in London, Canada. LUS clips obtained using a linear ultrasound probe were excluded, given the lack of linear clips in the original training set (<5%) [18] and the significant differences in acquisition physics (higher frequency and shallower penetrance) [26]. LUS clips were preprocessed into 3-second (s) segments and resized to 224 × 224 pixels for standardization of the model’s input. The total dataset consisted of 6413 s clips—557 with lung sliding and 84 without lung sliding. Detailed dataset characteristics are provided in Table 1.

For the purposes of this study, clips were assigned to the positive class if they exhibited absent lung sliding (i.e., suggestive of PTX) and to the negative class if they exhibited the presence of lung sliding and/or lung pulse (i.e., ruling out PTX).

**Figure 1:**
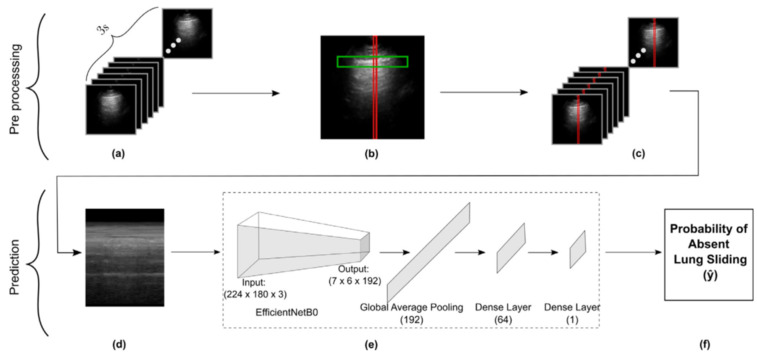
Schematic representation of our methods for data preprocessing through to M-mode creation and subsequent model development. (**a**) Frames in a 3 s LUS clip. (**b**) Vertical slice selection (red), restricted by pleural line ROI (green). (**c**) Vertical slicing across all frames. (**d**) Concatenating slices to form an M-mode image. (**e**) Obtaining the model’s prediction for the M-mode input image. (**f**) Final model output representing probability of absent lung sliding.

Images and texts extraneous to the ultrasound beam were removed from all clips using a dedicated deep learning tool (Automask, WaveBase, Inc., wavebase.ai accessed on 10 November 2023, Waterloo, ON, Canada). A previously trained pleural line object detection model isolated the pleurae of the first frame from each LUS clip, which guided M-mode extraction. This workflow is described in our previous work [18] and is summarized in Figure 1. To address the class imbalance disfavoring the absent lung sliding class, we upsampled examples during training experiments. Ten M-mode images were gathered from each absent lung sliding B-mode clip in our dataset during preprocessing. The M-mode image created from the column with the brightest pixel intensity was then selected for inclusion in the main dataset. The nine other images were sequestered into a separate pool of examples available to be randomly sampled from during training, as needed, to balance the class distribution. Note that the additional M-mode images vary from the original because they are produced using different columns with bright pixel intensities.

### 2.2. Model Fine-Tuning

We propose Threshold-Aware Accumulative Fine-Tuning (TAAFT), an approach to determine the minimum amount of data required to attain predefined performance metrics on datasets from different distributions. This approach allows us to (1) determine the minimum amount of external data that is required to attain targeted performance metrics and (2) evaluate our models on a more representative sample of the entire dataset (the variable-sized validation set), while maintaining the ability to directly compare each model’s performance on the same dataset (the fixed-size validation set).

In each TAAFT trial, the dataset was randomly divided (with a patient-wise split) into 2*k* folds that each contain approximately $\frac{1}{2k}$ of the dataset. Each unique patient only existed in either the training or the validation set. *k* ∈ {1, 2, 3, …} is a variable chosen based on how many differently sized training sets the developer would like to consider during a fine-tuning experiment. For the experiments in this study, we set *k* = 3. A minimal fixed-size validation set proportion of $\frac{1}{2}$ was chosen in this work, given the small size of the dataset to be used for fine-tuning and the sparseness of positive class examples. The mean characteristics of the folds used during all fine-tuning experiments described in this work are given in Table 2.

**Figure 2:**
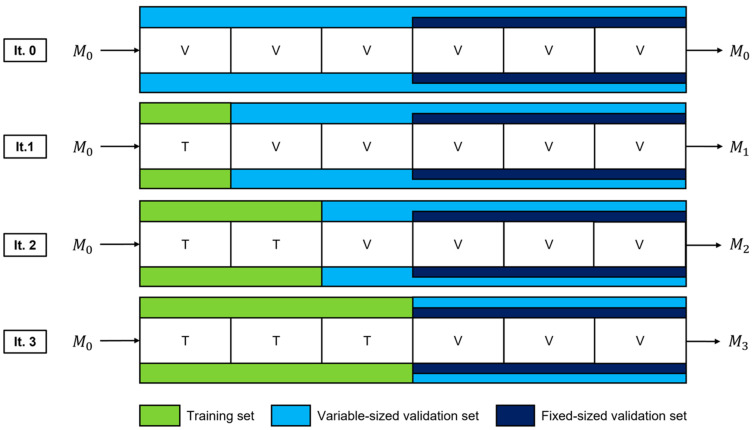
Dataset splits and fine-tuned models for a single trial of the TAAFT method. Data are incrementally added to the training set used for fine-tuning (green) and removed from the variable-sized validation set (light blue) while maintaining a fixed-size validation set (dark blue). This process continues until the two validation sets are the same. Three new models (M_1_, M_2_, and M_3_) are produced, each being fine-tuned using a different proportion of the dataset and evaluated on each validation set (variable-sized and fixed-size).

The training set is initially empty, and the original model [18], henceforth referred to as M_0_, is evaluated on both the entire dataset (the variable-sized validation set) and the union of the last k folds (the fixed-size validation set). Following iteration 0 (Figure 2), a fold is added to the training set (now $\frac{1}{2k}$ of the entire dataset) and removed from the variable-sized validation set (now $\frac{2k-1}{2k}$ of the entire dataset). This training set is used to fine-tune M_0_, and the performance of the resultant model (M_1_) is evaluated on both the variable-sized and the fixed-size validation set. This marks the completion of iteration 1, following which another fold is added to the training set (now $\frac{1}{k}$ of the entire dataset) and removed from the variable-sized validation set (now $\frac{k-1}{k}$ of the entire dataset). M_0_ is fine-tuned once again using the new, larger training set and its performance is evaluated on both validation sets. This process is repeated in subsequent iterations, until the training set encompasses half of the entire dataset and the variable-sized and fixed-size validation sets are identical. During a single TAAFT trial, *k* new models are produced that are each trained on a different proportion p_train_ of the dataset ($\frac{1}{2k}$, $\frac{1}{k}$, $\frac{3}{2k}$, …, $\frac{1}{2}$). Each model is then evaluated on the most representative sample of the dataset that is available (the variable-sized validation set) as well as a consistent (fixed-size) validation set to facilitate metric comparisons. Each fixed-size validation set within a single TAAFT trial contains the same images to maintain consistency and to limit variance, allowing for direct comparison between iterations.

To avoid favorable dataset splits biasing our results, 5 TAAFT trials with *k* = 3, each with different patient-wise folds, were completed on the external dataset. In total, 15 fine-tuned models, 5 at each training set proportion (p_train_ = $\frac{1}{6}$, $\frac{1}{3}$, and $\frac{1}{2}$), were produced during each fine-tuning experiment. An experiment was deemed successful if the mean trial-wise performance of at least one training proportion exceeded the predefined performance goals.

To fine-tune the final model, an optimal training set proportion was first selected by comparing the mean trial-wise sensitivity and specificity for absent lung sliding of a successful TAAFT experiment at that proportion to the average performance of the original model on locally obtained data. As in our previous work [18], sensitivity was chosen as the priority metric over specificity, given that a false negative (prediction of present lung sliding when no sliding is apparent) may lead to more patient harm compared to false positives. Specifically, we aimed to meet or exceed the lower bound of the mean ± standard deviation interval of our original cross validation experiment (0.901 sensitivity and 0.793 specificity [18]) on at least the variable-sized validation set, on average, at any training proportion. The smallest proportion meeting these predefined performance goals was then used to construct the final model’s training set. The remaining data were used for validation. The characteristics of the folds used to fine-tune and validate the final model are given in Table 2.

Prior to fine-tuning, each TAAFT training set underwent a random, patient-wise, 80/20 training/secondary validation set split. Data augmentation was applied to the training set as described in previous work [18]. Furthermore, to ensure the same class distribution as our original training set [18], absent lung sliding examples were upsampled from the sequestered pool of extra M-mode images. 

The model used in this study is a customized EfficientNetB0 [27], as described in previous work [18]. The TAAFT experiment was also evaluated on two additional common model architectures, ResNet18 [28] and MobileNetV3 [29]. A hyperparameter search was performed with learning rate, learning rate decay, drop out, and focal loss parameter. The code for all experiments along with hyperparameter search parameters is available via our GitHub repository.

### 2.3. Explainability and Error Analysis

The results of the final model were analyzed with respect to LUS metadata to identify performance differences that may guide future data collection. The subgroups considered included the machine vendor, probe type, imaging preset, depth, and institution. A chi-squared test for independence and a one-way analysis of variance (ANOVA) test were performed on each data characteristic to determine whether the correctness and error of the model’s predictions depend on that subgroup, respectively. Separate tests were performed on all ground-truth positive examples and all ground-truth negative examples in the dataset to study the effect of the subgroup on model sensitivity and specificity, respectively. Of the metric–subgroup combinations that met statistical significance (*p* ≤ 0.05) using the chi-squared test of independence, a within-subgroup fragility index was computed as a way to measure robustness and analyze within-subgroup dependencies.

We applied the Grad-CAM++ method [30] to visualize which components of the input M-mode images were most contributory to the model’s prediction. The results are conveyed by color on a saliency map, overlaid on the original input images. Blue and red regions correspond to the lowest and highest prediction importance, respectively.

False negative and false positive results from the final model were visually reviewed by clinicians to determine which features at the B-mode clip or the M-mode image level may be contributing to performance.

## 3. Results

**Figure 3:**
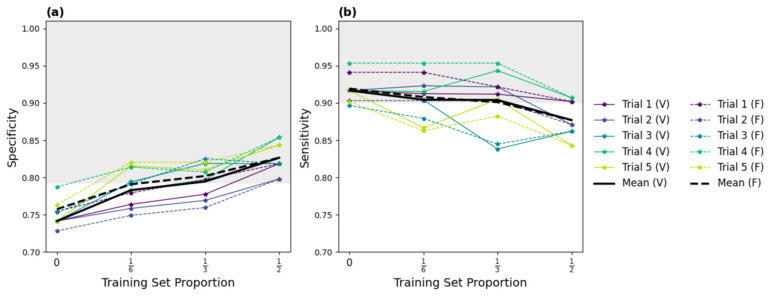
Specificity (**a**) and sensitivity (**b**) results of the successful five-trial TAAFT fine-tuning experiment. The mean (thick line) and individual (thin line) trial-wise metrics observed on the variable (solid) and fixed (dashed) sized validation sets at each training set proportion (p_train_) are shown. The predefined performance goals (sensitivity ≥ 0.901, specificity ≥ 0.793; shaded grey region) are met, on average, on the variable-sized validation set when M_0_ is fine-tuned on $\frac{1}{3}$ of the external dataset.

The results of our five-trial TAAFT experiments are displayed in Figure 3. Performance on the variable-sized validation sets (solid curves) and fixed-size validation sets (dashed curves) were comparable at each p_train_ ($\frac{1}{6}$, $\frac{1}{3}$, and $\frac{1}{2}$). Without any fine-tuning, the original model (p_train_ = 0) yielded an overall sensitivity of 0.917 and an overall specificity of 0.741 on the entire dataset. Fine-tuning improved detection of present lung sliding, with specificity increasing with the size of the training set (Figure 3a). Sensitivity remained nearly stable (Figure 3b) for smaller training proportions (p_train_ = $\frac{1}{6}$ and p_train_ = $\frac{1}{3}$), but not for larger training proportions (p_train_ = $\frac{1}{2}$), where a drop in performance on the positive class was observed. Fine-tuning on one-third of the external dataset yielded metrics exceeding our predefined performance goals, with mean specificity and sensitivity of 0.795 and 0.903, respectively, for absent lung sliding on the variable-sized validation set. The individual and mean trial-wise performance metrics yielded by the five models trained on one-third of the dataset on the variable-sized validation set are provided in Table 3. 

**Figure 4:**
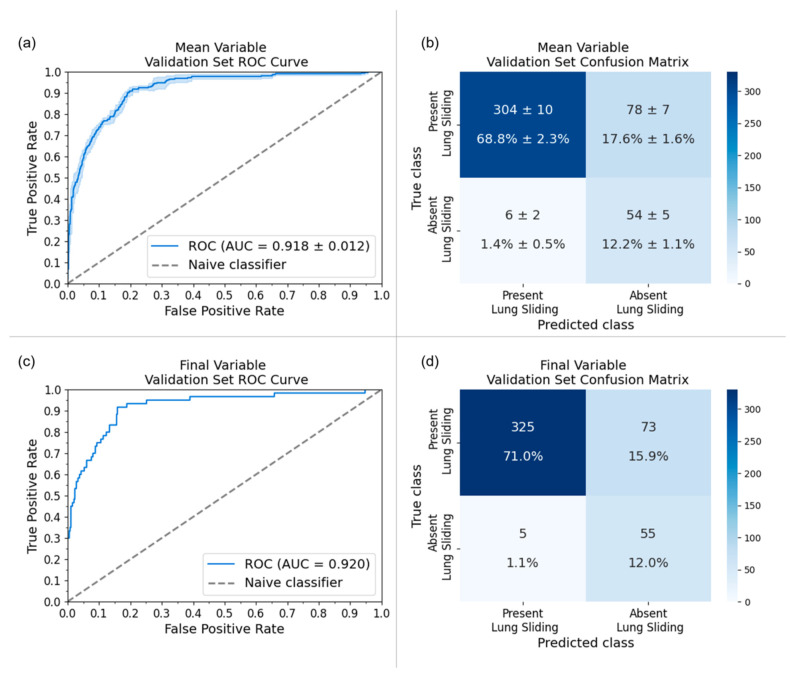
Receiver-operating characteristic (ROC) curves and confusion matrices for the five-trial TAAFT experiment (mean ± standard deviation) and final model performance on the variable-sized validation set at the optimal training proportion (p_train_ = $\frac{1}{3}$). (**a**) AUC of the five trial TAAFT experiment fine-tuning on $\frac{1}{3}$ of the external dataset with an average of 0.916 (standard deviation represented by the light blue outline) and (**b**) the corresponding confusion matrix. (**c**) AUC of inference of the final model yielded 0.920 and (**d**) the corresponding confusion matrix on its variable-sized validation set.

The final model was fine-tuned on one-third of the dataset using a different patient-wise split, which yielded a 0.917 sensitivity, 0.817 specificity, and 0.920 area under the receiver operator characteristic curve (AUC) on its variable-sized validation set. The final model’s performance was also evaluated on the original local holdout set [18] to assess for model drift from fine-tuning on external data. New M-mode images were generated from the original B-Mode LUS clips, and fine-tuning resulted in a 2.3% improvement in specificity (M_0_: 0.868, final model: 0.891) and a maintained (−0.7%) sensitivity (M_0_: 0.949, final model: 0.942) on the local holdout set. A full comparison of the original model (M_0_) performance and the final model performance on the original local holdout set and the final external variable-sized validation set can be found in Table 4. The receiver operator characteristic curves (ROC) and confusion matrices for both the TAAFT experiment and the final (external) variable-sized validation set are provided in Figure 4.

**Figure 5:**
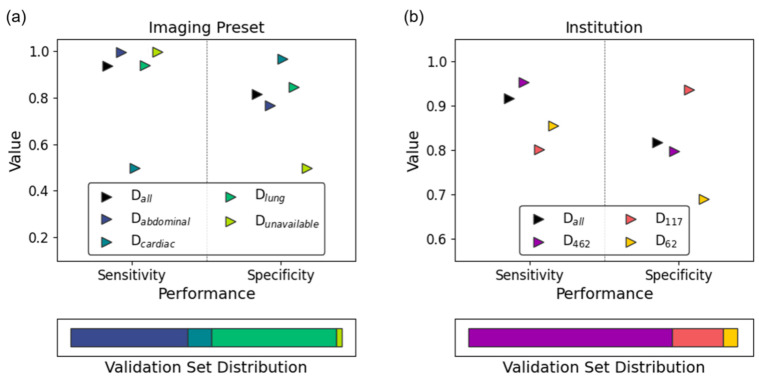
Subgroup analysis results of the final model on its variable-sized validation set. Sensitivity (circles) and specificity (squares) are stratified by (**a**) imaging preset and (**b**) institution. The validation set’s subgroup distribution is reflected in the bottom panel of each subplot.

Figure 5 and describe the subgroup-specific performance of our model. LUS clips obtained using the cardiac preset demonstrated excellent specificity with respect to absent lung sliding at the expense of notably reduced sensitivity (Figure 5a). Performance was poorer overall on data collected from Ottawa (Figure 5b, D_62_). The chi-squared test of independence identified exam preset (Figure 5a; *p* = 0.01), and institution (Figure 5b; *p* = 0.006) as significantly impacting model specificity. Within subgroup fragility indices highlighted clips acquired from cardiac presets, Chile (D_117_), and Mindray machines as predominantly contributing to these effects. Given that all D_117_ examples are acquired from Mindray machines, these results are highly correlated. The one-way ANOVA test identified the same subgroup–metric dependencies. Full details are provided in e-Appendix 5.

**Figure 6:**
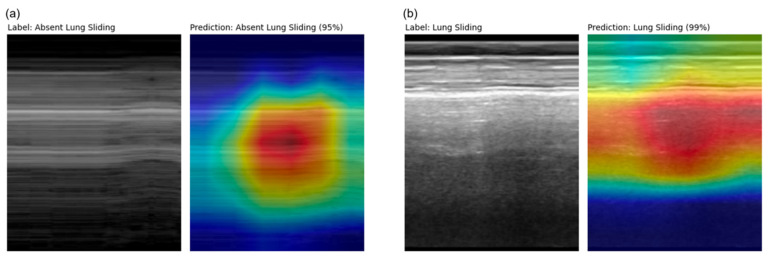
M-mode and corresponding Grad-CAM++ [23] saliency map images from a (**a**) true positive (D_462_) example and a (**b**) true negative (D_117_) example taken from the final model’s variable-sized validation set. Highly important features relating to model prediction are highlighted in red, which correspond to regions clinicians asses for lung sliding.

Saliency maps [30] for the final model’s variable-sized validation set were generated and revealed appropriate regions of prediction importance, centered at and below the pleural line where clinicians assess for the lung sliding artifact (Figure 6). This reflects the region on the ultrasound image in which clinicians make an assessment for lung sliding, enforcing biological correlation. 

Examination of false negative examples revealed LUS clips that (1) were inappropriately included in the dataset or (2) that were poorly acquired by the operator. One clip was noted to be inaccurately labelled as absent lung sliding when lung sliding was indeed present. A LUS clip containing a lung point and another of a pleural LUS view were also inappropriately included in the dataset, both of which met exclusion criteria based on our previous work [18]. Finally, two LUS clips demonstrated significant probe movement during image acquisition, which may mimic lung sliding, leading to incorrect model prediction.

**Figure 7:**
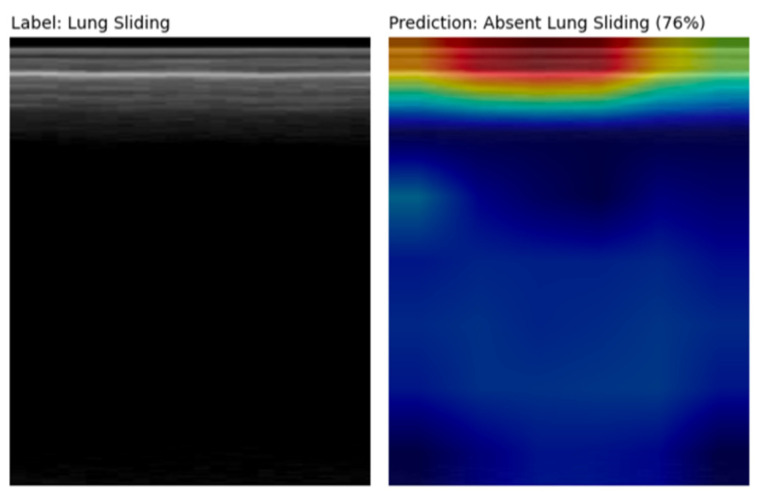
M-mode and corresponding Grad-CAM++ [27] saliency map image from a false positive prediction. The saliency map highlights the subcutaneous tissue above the pleural line that does not move with respiration, thus mimicking an absent lung sliding pattern. The significant depth at which this LUS clip was acquired likely contributed to the model’s incorrect prediction as well.

Several false positive examples had saliency maps that were focused above the pleural line on subcutaneous tissue that does not move with respiration, producing an M-mode that mimics absent lung sliding (Figure 7). Other false positive clips were noted to be acquired at greater depths.

## 4. Discussion

We report successful multicenter validation of our lung sliding classifier using a fine-tuning method that directly addresses the challenge of utilizing scarcely available external data to improve model generalizability. Optimally allocating limited external data for both fine-tuning and validation is crucial to balance learning new feature representations and evaluating model performance. Our methods provide a framework to evaluate and improve single-center-trained DL models for broader use and iterative enhancement.

Medical imaging datasets are small in comparison to traditional computer vision datasets [31], a challenge that is compounded in the field of LUS [32,33,34]. Although some DL models for medical image classification have demonstrated performance comparable to radiologists [35,36,37], it is estimated that only 6–13% of these studies include an external validation set [38,39,40]. Furthermore, 81% of these studies using external data demonstrate performance degradation [41]. Our TAAFT method addresses both issues of limited data availability and poor model generalizability in the context of external datasets, while allowing for flexibility in maximizing a desired performance metric. We demonstrate a 5.6% improvement in our model’s specificity while maintaining (−0.2%) sensitivity in a largely unbalanced dataset. This aligns with the clinical emphasis on the sensitivity of PTX, as missing this lethal diagnosis could delay life-saving procedures. We found that fine-tuning using one-third of our particular dataset yielded the best results. However, the TAAFT method can be customized using varying values of *k* to tailor this method to other applications depending on the amount of data available and the priority metric (sensitivity vs. specificity). Examination of false negative predictions revealed image acquisition-related issues affecting image preprocessing, which may reflect an expected degree of operator dependency with point-of-care imaging during real-world use. The pronounced class imbalance disfavoring the positive class may also stunt improvements in sensitivity. We employed several mitigating factors, such as upsampling, data augmentation, and use of a class-weighted loss function. However, there should be future efforts to obtain more examples to balance the class representation. Saliency maps confirmed biological plausibility for model predictions by highlighting anatomically relevant regions. Finally, binary cross-entropy often struggles with imbalanced datasets [42] and further investigation of more adaptable loss functions under these circumstances is warranted.

Presently, DL studies in the medical domain only provide a static representation of model performance without offering strategies for further improvement. Machine Learning Operations (MLOps) provides a framework for continual quality assurance and model improvement [43]. A clinical parallel can be made with the PDSA cycle in the Quality Improvement methodology [44], where feedback from workflow adjustments informs system changes in a cyclic manner. Currently, MLOps workflows are primarily deployed in industry applications, such as automated defect inspection in factory settings [45]. Tartaisco et al. [46] have prototyped a cloud-based machine continual learning framework for automated detection of valvular disease using heart sounds. Our work demonstrates a framework for MLOps practices and a data-centric approach for identifying areas for iterative model improvement in medical image classification. Metadata collection facilitated analysis and identification of poorly performing subgroups, which can serve to direct targeted data collection and further fine-tuning to better incorporate poorly represented features. This information can guide implementation of upstream metadata-aware preprocessing methods to optimize model inputs. Continual learning methods incorporating MLOps principles can be used to defend against biases from small datasets that do not represent features found in other datasets.

The success of our fine-tuning approach has yielded an absent lung sliding detection model with enriched diagnostic performance and generalizability. Such a system could be paired with portable ultrasound hardware to permit non-traditional users of ultrasound (e.g., paramedics, respiratory therapists, and military personnel) to assess for a life-threatening PTX virtually anywhere. With the maturation of wearable ultrasound devices [47,48], eventual automated and real-time monitoring of PTXs at the bedside is also conceivable.

## 5. Limitations and Future Directions

We acknowledge there are limitations to our work. We attempted to mitigate confounding variables in our heterogeneous data by describing the meta-data. However, further efforts to collect clinical descriptors such as diagnoses may strengthen correlations between model predictions and ground truth labels. Additionally, while we described several ultrasound-specific variables such as probe preset and manufacturer, we excluded all examples of linear probes. Targeted collection and fine-tuning using LUS images captured using linear probes will be prudent in further improving the generalizability of our model. Lastly, our model’s performance was only assessed on retrospective data. As such, future efforts should move towards prospective validation with comparison to expert annotations [49].

Future work by our group will investigate additional techniques to combat against poor generalizability of DL models in the setting of scarcely available labelled medical data. One area of interest is using self-supervised pretraining, which has demonstrated promise in improving task performance compared to full supervised learning for multiple medical imaging modalities including ultrasound [50]. This technique is particularly useful in the case when unlabeled examples vastly outnumber labelled examples. Preliminary studies have demonstrated improved performance, generalizability to external datasets, and inference time [51,52]. This technique could be leveraged to capture hardware variances amongst external centers, including different ultrasound manufacturers, probes, and presets. Deliberate focus on improving model generalizability has ethical implications, such as ensuring proper representation of patients from various demographic backgrounds. Striving to collect and annotate metadata will provide crucial transparency in DL models to identify and work towards eliminating model bias.

## 6. Conclusions

An absent lung sliding detection model was successfully validated on multi-institutional data. We improved the performance and generalizability of our EfficientNetB0 lung sliding classifier by employing our proposed TAAFT method to fine-tune with one-third of the available dataset. Poorly performing subgroups were also identified via subgroup analyses and serve as targets for future data collection. This work demonstrates the benefits of data-centric practices and collaboration between clinicians and engineers for iterative model improvement.

## Figures and Tables

**Table 1:** Ultrasound data characteristics between all data sources. The characteristics of the original, locally sourced holdout set from our previous work are also provided for comparison.

		Local Data		External Data							
				D_462_		D_117_		D_62_		D_all_	
		Sliding	Absent	Sliding	Absent	Sliding	Absent	Sliding	Absent	Sliding	Absent
Patients	By source	122		163		53		22		**238**	
	By class	88	36	154	36	48	6	21	7	**223**	**49**
Sex	Male	**46** **(37%)**	**25** **(20%)**	75 (39%)	22 (12%)	25 (46%)	5 (9%)	9 (32%)	3 (11%)	**109** **(40%)**	**30** **(11%)**
	Female	**42** **(34%)**	**11** **(9%)**	47 (25%)	12 (6%)	23 (43%)	1 (2%)	9 (32%)	2 (7%)	**79** **(29%)**	**15** **(5%)**
	Unavailable	**0** **(0%)**	**0** **(0%)**	32 (17%)	2 (1%)	0 (0%)	0 (0%)	3 (10%)	2 (7%)	**35** **(13%)**	**4** **(1%)**
Age	Mean (std)	**60.0** **(17.3%)**	**64.9** **(13.9%)**	56.4 (16.4%)	58.5 (13.1%)	55.9 (22.0%)	43.3 (20.8%)	56.8 (16.7%)	50.5 (19.1%)	**56.3** **(18.0%)**	**55.5** **(16.1%)**
	Unavailable	**0** **(0%)**	**0** **(0%)**	32 (17%)	2 (1%)	0 (0%)	0 (0%)	2 (7%)	2 (7%)	**34** **(12%)**	**4** **(1%)**
Clips	By source	**540**		462		117		62		**641**	
	By class	**402** **(74%)**	**138** **(26%)**	404 (88%)	58 (12%)	107 (91%)	10 (9%)	46 (74%)	16 (26%)	**557** **(87%)**	**84** **(13%)**
Machine Vendors	Phillips	**0** **(0%)**	**2** **(0%)**	0 (0%)	0 (0%)	0 (0%)	0 (0%)	24 (39%)	9 (15%)	**24** **(4%)**	**9** **(1%)**
	Sonosite	**395** **(73%)**	**96** **(18%)**	398 (86%)	58 (13%)	0 (0%)	0 (0%)	13 (21%)	0 (0%)	**411** **(64%)**	**58** **(9%)**
	Mindray	**7** **(1%)**	**40** **(7%)**	0 (0%)	0 (0%)	107 (91%)	10 (9%)	6 (10%)	5 (8%)	**113** **(18%)**	**16** **(2%)**
	Unavailable	**0** **(0%)**	**0** **(0%)**	6 (1%)	0 (0%)	0 (0%)	0 (0%)	3 (5%)	2 (3%)	**9** **(1%)**	**2** **(0%)**
Probe	Phased Array	**366** **(68%)**	**118** **(22%)**	337 (73%)	52 (11%)	65 (56%)	3 (3%)	20 (32%)	1 (2%)	**422** **(66%)**	**56** **(9%)**
	Curved Linear	**32** **(6%)**	**14** **(3%)**	67 (15%)	6 (1%)	42 (36%)	7 (6%)	26 (42%)	15 (24%)	**135** **(21%)**	**28** **(4%)**
Location	ED	**122** **(23%)**	**12** **(2%)**	0 (0%)	0 (0%)	107 (91%)	10 (9%)	24 (39%)	13 (21%)	**131** **(20%)**	**23** **(4%)**
	ICU	**274** **(51%)**	**124** **(23%)**	401 (87%)	58 (13%)	0 (0%)	0 (0%)	19 (31%)	1 (2%)	**420** **(65%)**	**59** **(9%)**
	Unavailable	**0** **(0%)**	**0** **(0%)**	3 (1%)	0 (0%)	0 (0%)	0 (0%)	3 (5%)	2 (3%)	**6** **(1%)**	**2** **(0%)**
Imaging Preset	Abdominal	**373** **(69%)**	**104** **(19%)**	194 (42%)	21 (5%)	45 (38%)	4 (3%)	20 (32%)	13 (21%)	**259** **(41%)**	**38** **(6%)**
	Cardiac	**14** **(3%)**	**4** **(1%)**	23 (5%)	0 (0%)	20 (17%)	2 (2%)	4 (6%)	0 (0%)	**47** **(7%)**	**2** **(0%)**
	Lung	**11** **(2%)**	**24** **(4%)**	178 (39%)	37 (8%)	42 (36%)	4 (3%)	16 (26%)	1 (2%)	**236** **(37%)**	**42** **(7%)**
	Unavailable	**0** **(0%)**	**0** **(0%)**	9 (2%)	0 (0%)	0 (0%)	0 (0%)	6 (9%)	2 (3%)	**15** **(2%)**	**2** **(0%)**
Depth	<6 cm	**14** **(3%)**	**8** **(1%)**	4 (1%)	0 (0%)	2 (2%)	0 (0%)	4 (6%)	0 (0%)	**10** **(2%)**	**0** **(0%)**
	6–20 cm	**382** **(71%)**	**130** **(24%)**	395 (85%)	58 (13%)	104 (89%)	10 (9%)	40 (65%)	16 (26%)	**539** **(84%)**	**84** **(13%)**
	>20 cm	**6** **(1%)**	**0** **(0%)**	5 (1%)	0 (0%)	1 (1%)	0 (0%)	2 (3%)	0 (0%)	**8** **(1%)**	**0** **(0%)**

**Table 2:** Summary characteristics of the folds used (a) during the 5-trial TAAFT experiment (mean ± standard deviation) and (b) for fine-tuning the final model. The individual folds used in each of the TAAFT trials comprising (a) are detailed in e-Table 1.

	Data	Fold 1		Fold 2		Fold 3		Fold 4		Fold 5		Fold 6	
		Sliding	Absent	Sliding	Absent	Sliding	Absent	Sliding	Absent	Sliding	Absent	Sliding	Absent
(a)	Patients	36 ± 3 (83 ± 5%)	7 ± 2 (17 ± 5%)	38 ± 3 (84 ± 2%)	7 ± 1 (16 ± 2%)	37 ± 2 (82 ± 3%)	8 ± 2 (18 ± 3%)	36 ± 3 (78 ± 4%)	10 ± 3 (22 ± 4%)	39 ± 4 (86 ± 2%)	6 ± 2 (14 ± 2%)	37 ± 2 (80 ± 4%)	10 ± 2 (20 ± 4%)
	Clips	85 ± 6 (88 ± 5%)	11 ± 5 (12 ± 5%)	89 ± 6 (88 ± 4%)	12 ± 5 (12 ± 4%)	98 ± 8 (88 ± 3%)	12 ± 4 (12 ± 3%)	94 ± 6 (84 ± 5%)	18 ± 7 (16 ± 5%)	100 ± 15 (89 ± 2%)	12 ± 4 (11 ± 2%)	91 ± 8 (84 ± 5%)	16 ± 5 (16 ± 5%)
(b)	Patients	30 (80%)	9 (20%)	37 (80%)	9 (20%)	42 (81%)	10 (19%)	35 (90%)	4 (10%)	36 (82%)	8 (18%)	38 (81%)	9 (19%)
	Clips	92 (88%)	13 (12%)	67 (86%)	11 (14%)	108 (87%)	16 (13%)	71 (91%)	7 (9%)	106 (82%)	24 (18%)	113 (90%)	13 (10%)

**Table 3:** Metrics for a successful fine-tuning experiment with five TAAFT trials, as computed on the variable-sized validation set at the optimal training proportion ($\frac{1}{3}$). Mean sensitivity and specificity exceeded the predefined performance goals (sensitivity 0.901, specificity 0.793).

Trial	Sensitivity	Specificity	AUC	Accuracy
1	0.912	0.777	0.919	0.798
2	0.922	0.769	0.911	0.787
3	0.838	0.819	0.908	0.822
4	0.943	0.797	0.942	0.814
5	0.905	0.810	0.912	0.824
Mean	0.903	0.795	0.918	0.809
(STD)	(0.035)	(0.019)	(0.012)	(0.014)

**Table 4:** Comparing the performance of the original (non-fine-tuned) model (M_0_) and final (fine-tuned) model on the final variable-sized external validation set and the original local holdout set [9]. The final model’s sensitivity and specificity for absent lung sliding, as evaluated on the external validation set exceeded the predefined performance goals (sensitivity 0.901, specificity 0.793).

Dataset	Model	Sensitivity	Specificity	AUC	Accuracy
External Validation	Final	0.917	0.817	0.920	0.830
	M_0_	0.919	0.761	0.914	0.782
Local Holdout	Final	0.942	0.891	0.974	0.904
	M_0_	0.949	0.868	0.973	0.889

## Data Availability

The datasets presented are not readily available due to privacy restrictions but may be able to be made available in the future.
